# Ensemble learning for fetal ultrasound and maternal–fetal data to predict mode of delivery after labor induction

**DOI:** 10.1038/s41598-024-65394-6

**Published:** 2024-07-03

**Authors:** Iolanda Ferreira, Joana Simões, Beatriz Pereira, João Correia, Ana Luísa Areia

**Affiliations:** 1 Faculty of Medicine of University of Coimbra, Obstetrics Department, University and Hospitalar Centre of Coimbra, Coimbra, Portugal; 2 Maternidade Doutor Daniel de Matos, R. Miguel Torga, 3030-165 Coimbra, Portugal; 3 Department of Informatics Engineering, Centre for Informatics and Systems of the University of Coimbra, University of Coimbra, Coimbra, Portugal; 4 Department of Physics, University of Coimbra, Coimbra, Portugal

**Keywords:** Outcomes research, Ultrasonography

## Abstract

Providing adequate counseling on mode of delivery after induction of labor (IOL) is of utmost importance. Various AI algorithms have been developed for this purpose, but rely on maternal–fetal data, not including ultrasound (US) imaging. We used retrospectively collected clinical data from 808 subjects submitted to IOL, totaling 2024 US images, to train AI models to predict vaginal delivery (VD) and cesarean section (CS) outcomes after IOL. The best overall model used only clinical data (F1-score: 0.736; positive predictive value (PPV): 0.734). The imaging models employed fetal head, abdomen and femur US images, showing limited discriminative results. The best model used femur images (F1-score: 0.594; PPV: 0.580). Consequently, we constructed ensemble models to test whether US imaging could enhance the clinical data model. The best ensemble model included clinical data and US femur images (F1-score: 0.689; PPV: 0.693), presenting a false positive and false negative interesting trade-off. The model accurately predicted CS on 4 additional cases, despite misclassifying 20 additional VD, resulting in a 6.0% decrease in average accuracy compared to the clinical data model. Hence, integrating US imaging into the latter model can be a new development in assisting mode of delivery counseling.

## Introduction

Induction of labor (IOL) is a common obstetric procedure that involves artificially initiating uterine contractions to start labor and delivery^1^. IOL rates are increasing worldwide, particularly in developed countries, accounting for 25% of deliveries in the UK and US^2,3^. IOL can lead to a cesarean section (CS), although it does not increase its rate^4^. Efforts are being made to reduce unnecessary CS and achieve suitable rates, as recommended by the World Health Organization (WHO)^5^. Factors such as Bishop score, parity, previous CS, maternal body mass index and weight gain during pregnancy are known to play a crucial role in these rates^6,7^.

Obstetric ultrasound (US) is an essential imaging modality for fetal monitoring during pregnancy, which provides an economic and non-invasive way of assessing fetal organ development and growth^8^. Ultrasound assessment of fetal biometry and growth is performed by obtaining measures of the fetal head circumference (HC), biparietal diameter (BPD), abdominal circumference (AC), and femur length (FL)^9,10^. In clinical practice, fetal biometry, fetal weight estimation, and/or Doppler blood flow are frequently used as biomarkers for fetal growth disorder screening, performed following international guidelines^11,12^. These images obey protocols that produce comparable images acquired in specific planes, to be useful for diagnosis, reduce intra- and interobserver variability and allow measuring of particular structures^8^.

Artificial Intelligence (AI) is the branch of computer science that focuses on creating systems capable of performing tasks that require some level of intelligence such as learning, reasoning, problem-solving, perception, and decision-making^10^. Machine learning (ML), a subset of AI, uses algorithms that enable computers to learn from data and improve their performance without explicitly programming them^13^. AI and ML are gaining popularity in healthcare due to their ability to analyze complex data structures and patterns, such as electronic health records and medical images, and create prediction models, ultimately improving individual health outcomes^14^. In the specific field of medical imaging, including US, ML has shown several advancements with the employment of deep learning (DL) models^10,15^. They excel in image recognition, classification, detection, and segmentation, surpassing human capabilities^13,15,16^. DL models can use supervised or unsupervised learning approaches. Supervised models use labeled data during training, followed by unlabeled data testing. Unlike traditional software, which use preset logic rules, DL models use raw data as input^10,17^. DL models are being proposed to support sonographers in US, overcoming issues like subjectivity and interobserver variability. They can also reduce examination times and tutor young doctors. It is important to note, however, that AI is not a replacement for human healthcare professionals, but rather a human support decision-making tool^18,19^.

The success of DL models depends on convolutional neural networks (CNNs), that are able to extract patterns automatically from images and use them to learn to map the input data (i.e. US images) and output data (a label, such as vaginal delivery (VD) and CS)^15,18^. Moreover we can use transfer learning to adapt existing CNN architectures that were trained in large, labeled datasets (i.e., ImageNet) to reduce the amount of data and computation needed to produce accurate models, thereby overcoming the occasionally observed scarcity of large volumes of labeled data in the imaging medical field^13,17^.

AI methodology has been used in obstetrics and gynecology to evaluate adnexal masses, endometrial cancer risk, pelvic organ function, breast lesions, and predict fetal outcomes, improving prenatal diagnosis^13,18^. CNNs are used in obstetric US for fetal weight estimation by measuring fetal biometry, identification of normal and abnormal anatomy, and detection and localization of structures and standard planes. This has already instituted clinical applications in fetal imaging, including echocardiography and neurosonography^15,20^. Recent literature findings by Kim et al.’s^21^ study group have developed a DL algorithm for automated measurement of BPD and HC, improving localization of fetal head shapes and caliper placement in later gestational ages. Rizzo et al.^22^ developed a DL software to automate fetal central nervous system assessment measurements, reducing examination time and reliance on fetal position and 2D-ultrasound expertise. Intrapartum ultrasound has been reported as a valuable tool for providing accurate and reproducible labor progression findings, including fetal head position, station, and flexion, crucial for labor management. In this context, Ghi et al.^23^ proposed a DL implementation for assessing fetal occiput position before vaginal delivery, achieving an accuracy of 90.4% in recognizing the fetal head position.

Several international societies have suggested that US evaluation may help predict delivery mode after IOL, using evaluations such as cervical length and posterior cervical angle measurements^24^. However, to our knowledge, no studies have considered using fetal biometry images for this purpose, although obstetricians rely on US fetal weight estimation to guide delivery counseling. Therefore, we hypothesized that DL could aid in mode of delivery prediction after IOL by analyzing maternal and fetal data obtained from electronic medical records (EMR) and fetal US biometry images. Although several maternal characteristics have been related with an increased risk of CS^25^ this relationship has been harder to prove with fetal US features. Since US fetal biometry evaluation is influenced by experience, maternal habitus and fetal position, among other factors^10^, potential errors significantly impair our ability to detect anomalous fetal growth^11^. That is why the International Society of Ultrasound in Obstetrics and Gynecology (ISUOG) recommends that fetal biometry ought to be just one part of our fetal growth screening process, and that a combined approach using other clinical, biological and/or imaging markers may also be applied^11^.

As such, the main objectives of this study are to develop and test ML and DL models for predicting mode of delivery (VD or CS) after IOL using tabular and US fetal imaging data in standard biometry planes. Secondary objectives are to create an ensemble of the best performing models and calculate and compare their diagnostic predictive accuracy.

## Results

### Tabular data

From January 2018 and December 2021, 808 patients with singleton vertex pregnancies were included in our longitudinal retrospective study, with 563 (69.7%) patients culminating in VD and 245 (30.3%) categorized as unplanned CS. The participants’ average age was 32.2 years [18–47 ± 5.7]. Demographic features and maternal and neonatal outcomes are shown in Table 1. Comparison between the two delivery modes showed significant differences in terms of age, height, body mass index (BMI) and parity, with more parous women in the VD group (37.8% vs 32.2%; *p* < 0.001). Women in the CS group were older, shorter, had a higher BMI and heavier babies at birth (*p* < 0.001). Other characteristics, such as gestational diabetes, 5-min Apgar scores ≤ 7, and neonatal intensive care unit admission rates were similar between groups. Mean gestational age (GA) on third trimester US was 30.9 ± 0.94 weeks [27–32 weeks]. The mean fetal biometry measures were, respectively: HC 290.7 ± 12.7 mm [245.8–326.6 mm], BPD 81.1 ± 4.0 mm [57.0–91.1 mm], AC 278.6 ± 15.1 mm [215.0–325.6 mm], FL 60.0 ± 2.9 mm [47.1–68.0 mm] and estimated fetal weight (EFW) 1848.8 ± 247.9 mm [903.0–2610.0 mm]. The recommended Hadlock formula was used to calculate EFW^9^. All these measurements were significantly different between both groups, except for FL, which showed similar values (see Table 1).

**Table 1:** Demographic, induction of labour and delivery outcome data for women who delivered vaginally vs. unplanned caesarean.

	Vaginal delivery	Cesarean delivery	*p*-value
Demographic data			
Maternal age in years (mean, SD)	31.8 (± 5.7)	33.1 (± 5.7)	**0.003**
Maternal height in cm (mean, SD)	163.7 (± 5.9)	161.7 (± 6.2)	** < 0.001**
Nuliparity (n, %)	294 (62.2%)	166 (67.8%)	** < 0.001**
Previous CS (n, %)	39 (6.9%)	56 (23.2%)	** < 0.001**
Body mass index (kg/m^2^, SD)^a^	30.6 (± 4.9)	32.2 (± 5.3)	** < 0.001**
US data			
EFW in grams (mean, SD)	1832.9 (± 247.3)	1885.3 (± 245.9)	**0.008**
HC in mm (mean, SD)	289.9 (± 12.6)	292.6 (± 12.7)	**0.006**
BPD in mm (mean, SD)	80.8 (± 4.1)	81.7 (± 3.8)	**0.005**
AC in mm (mean, SD)	277.4 (± 15.2)	281.3 (± 14.6)	**0.001**
FL in mm (mean, SD)	60.0 (± 2.9)	60.0 (± 2.8)	0.935
IOL data			
IOL method (n, %)^b^			** < 0.001**
Misoprostol	279 (49.6%)	73 (29.8%)	** < 0.001**
Dinoprostone	257 (45.6%)	162 (66.1%)	** < 0.001**
Foley catheter	12 (2.1%)	6 (2.4%)	0.983
Oxitocin	9 (1.6%)	3 (1.2%)	0.930
Gestational age at IOL (mean, SD)	39.9 (± 1.1)	40.1 (± 1.0)	**0.019**
Indication for labour induction			
Post term pregnancy (n, %)	257 (45.6%)	131 (53.5%)	0.049
Premature rupture of membranes (n, %)	35 (6.2%)	20 (8.2%)	0.391
Gestational diabetes mellitus (n, %)	50 (8.9%)	17 (6.9%)	0.435
Intrahepatic cholestasis (n, %)	3 (0.5%)	1 (0.4%)	1.0
Oligohydramnios (n, %)	33 (5.9%)	12 (4.9%)	0.702
Hypertensive disorders (n, %)	14 (2.5%)	13 (5.3%)	0.066
Preeclampsia (n, %)	18 (3.2%)	9 (3.7%)	0.894
Maternal pathology (n, %)	34 (6.0%)	11 (4.5%)	0.474
Fetal pathology (n, %)	8 (1.4%)	2 (0.8%)	0.713
Assisted reproduction (n, %)	7 (1.2%)	8 (3.3%)	0.094
Other (n, %)	104 (18.5%)	21 (8.6%)	**0.01**
BISHOP			** < 0.001**
0–3 (n, %)	425 (75.5%)	225 (91.8%)	** < 0.001**
4–5 (n, %)	123 (21.8%)	19 (7.8%)	** < 0.001**
6 (n, %)	15 (2.7%)	1 (0.4%)	0.066
Time until delivery h (mean, SD)	20.1 (± 13.7)	28.9 (± 16.8)	** < 0.001**
Delivery data			
Mode of delivery (%)	563 (69.7%)	245 (30.3%)	
Eutocic delivery	308 (54.7%)	–	–
Vaginal instrumental delivery	255 (45.3%)	–	–
Cesarean section indications			
FHR abnormality	–	74 (30.2%)	–
Failed induction	–	59 (24.1%)	–
Labour dystocia	–	105 (42.9%)	–
Neonatal outcome data			
Neonatal birthweight (grams, SD)	3330.2 (± 418.3)	3498.7 (± 428.7)	** < 0.001**
5-min Apgar Score ≤ 7 (%)	7 (1.2%)	2 (0.8%)	0.867
Neonatal intensive care admission (%)	10 (1.8%)	4 (1.6%)	1.0

^a^There were 19 missing values on the VD group and 15 missing values in the CS group concerning body mass index.

^b^There were 6 missing values on the VD group and 1 missing value in the CS group concerning IOL method.

Significant values are in bold.

Mean GA at IOL was similar between groups (39.9 vs 40.1 weeks). Dinoprostone was more frequently used in the CS group (66.1%), and the contrary was true for misoprostol (49.6%). Also, 91.8% of pregnant women in the CS group presented significantly lower Bishop scores (≤ 3) before IOL. IOL indications did not differ between groups. Time to delivery was significantly longer (20.1 versus 28.9 h) in the CS group. A third of CS were due to non-reassuring fetal heart rate (30.2%), while the majority (67%) corresponded to “failed induction/labor dystocia” (see Table 1).

**Figure 1:**
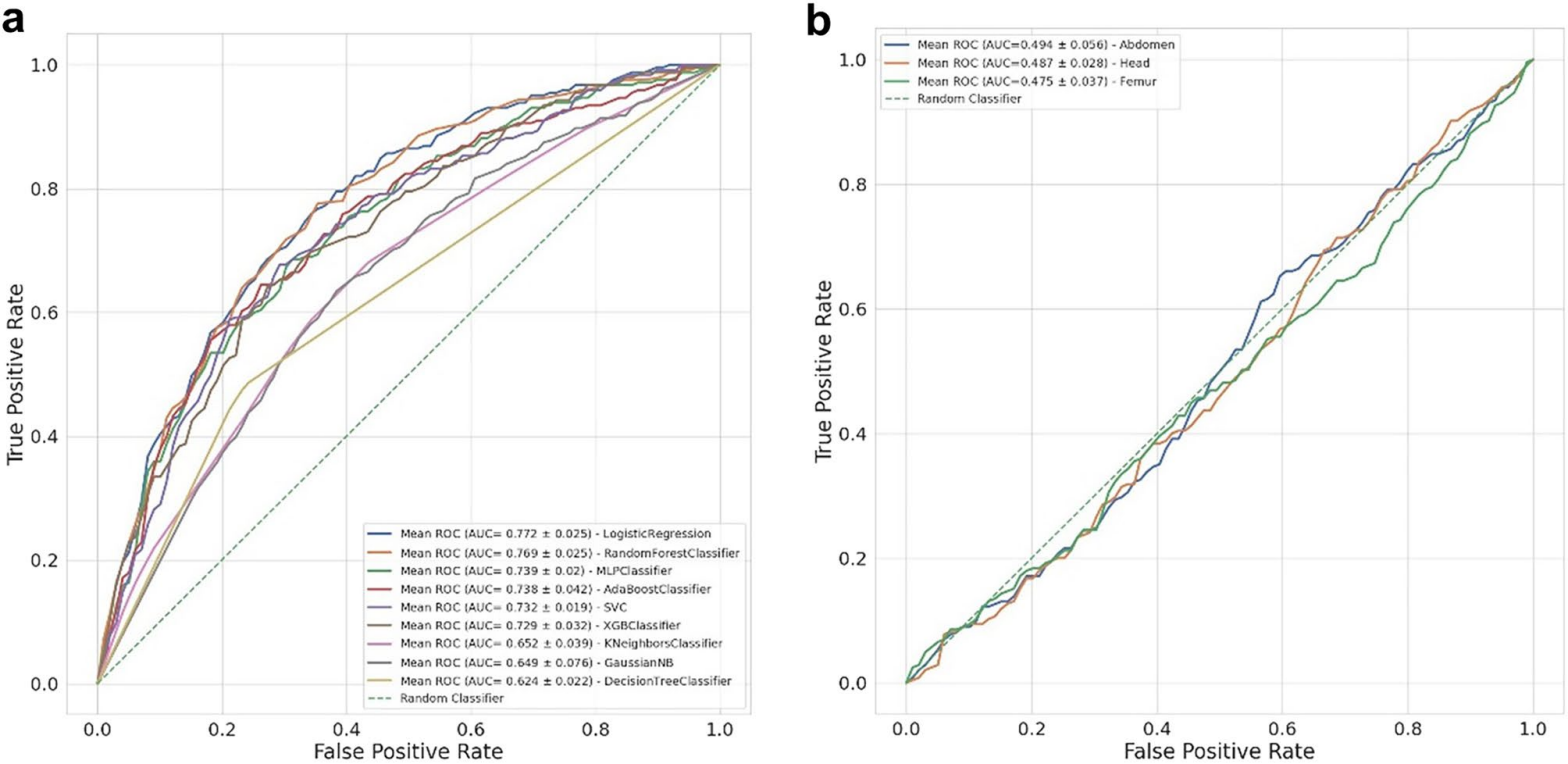
The ROCs for prediction of mode of delivery for tabular data (**a**) and image-based data (**b**). ROC, receiver operating characteristic.

**Figure 2:**
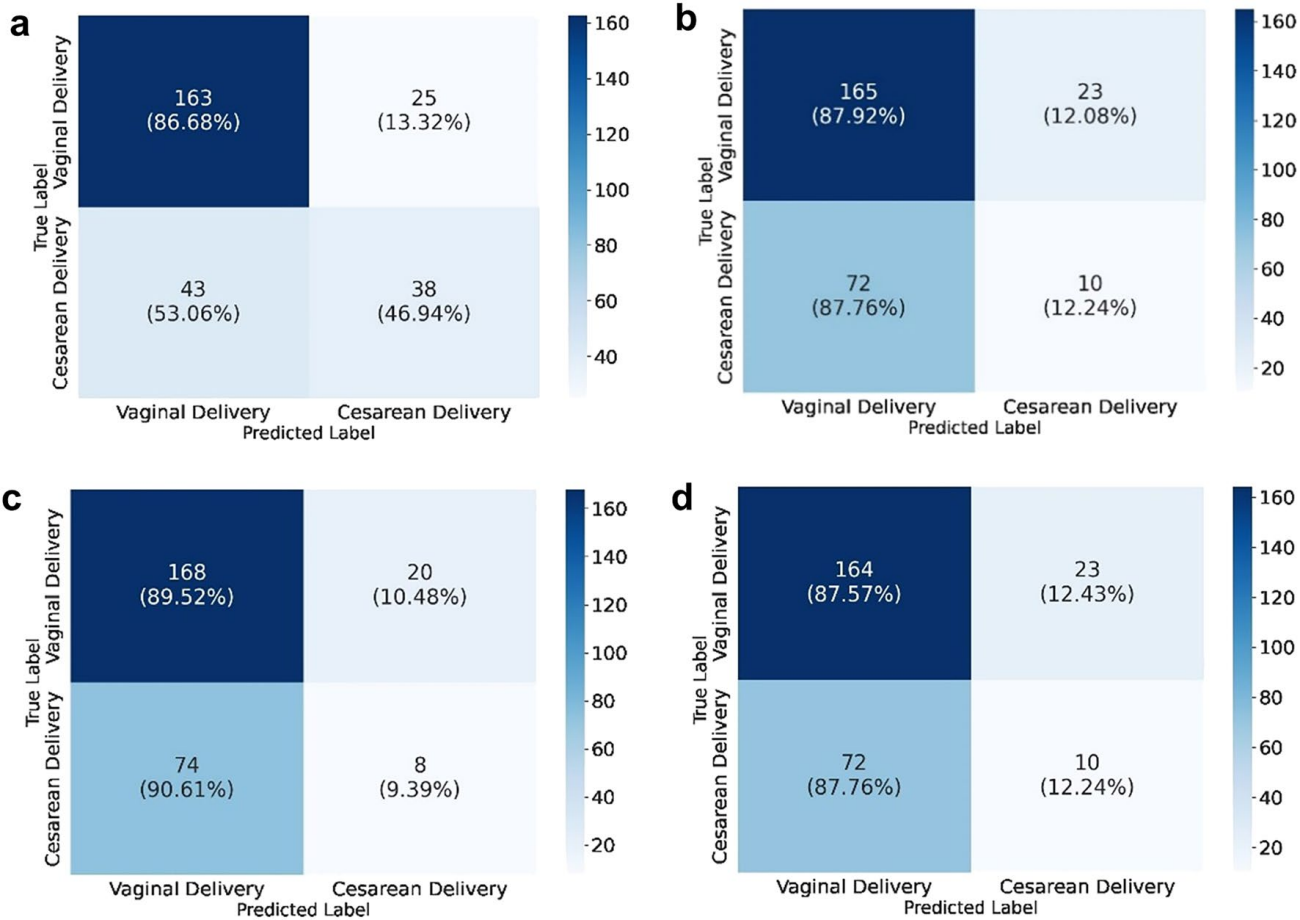
Confusion matrices on (**a**) the best tabular data model AdaBoost and (**b**) the best image-based model of the femur, (**c**) head and (**d**) abdomen are shown. Confusion matrix depicting in reading order from left to right, top to bottom: true-negative, false-negative, false-positive and true-positive rates.

Figures 1 and 2 and Table 2 show the tabular data models’ performance in predicting CS likelihood. These models take into consideration maternal clinical data as well as fetal information provided by the third trimester US. The best performing model was selected for further interpretation due to its superior positive predictive value (PPV) and F1-score weighted, meaning that it has the best ratio between true positives (TP) and false positives (FP). The rationale for this choice is because a mode of delivery prediction model should detect as many CS as possible—true positives—while avoiding misclassifying a VD as a CS—false positives. All models showed good predictive performance, with F1-scores ranging from 0.59 to 0.74. The AdaBoost model presented a high predictive power (F1-score = 0.736 ± 0.024 and PPV 0.734 ± 0.024) and accurately predicted 86.7% VD and 46.9% of CS, corresponding to a 13.3% FP rate and 53.1% FN rate and an overall accuracy of 74.7% (201/269; see Fig. 2a). All results were obtained using cross-validation, which ensures that the model generalizes from training to test data not previously seen while evaluating all the dataset.

**Table 2:** Performance metrics of the DL models on the datasets.

Model	AUROC	F1-score	PPV	NPV	Sensitivity	Specificity
Tabular data models						
AdaBoost	0.738 ± 0.051	0.736 ± 0.024	0.734 ± 0.024	0.790 ± 0.019	0.746 ± 0.022	0.867 ± 0.019
Logistic regression	0.772 ± 0.031	0.731 ± 0.039	0.728 ± 0.040	0.789 ± 0.027	0.740 ± 0.036	0.856 ± 0.030
MLP	0.739 ± 0.024	0.730 ± 0.010	0.727 ± 0.011	0.794 ± 0.013	0.736 ± 0.007	0.840 ± 0.011
RandomForest	0.769 ± 0.031	0.725 ± 0.027	0.730 ± 0.027	0.772 ± 0.020	0.746 ± 0.021	0.902 ± 0.016
XGBoost	0.729 ± 0.039	0.705 ± 0.015	0.701 ± 0.016	0.775 ± 0.014	0.713 ± 0.011	0.829 ± 0.006
SVC	0.732 ± 0.023	0.587 ± 0.003	0.727 ± 0.112	0.700 ± 0.001	0.697 ± 0.010	0.988 ± 0.021
Image data models						
Inception femur	0.485 ± 0.019	0.605 ± 0.015	0.596 ± 0.017	0.703 ± 0.006	0.668 ± 0.003	0.908 ± 0.022
Inception head	0.492 ± 0.036	0.584 ± 0.028	0.570 ± 0.043	0.693 ± 0.013	0.655 ± 0.036	0.904 ± 0.052
Inception abdomen	0.487 ± 0.034	0.584 ± 0.017	0.563 ± 0.025	0.692 ± 0.009	0.645 ± 0.015	0.883 ± 0.011
Xception femur	0.505 ± 0.075	0.597 ± 0.035	0.573 ± 0.053	0.700 ± 0.016	0.653 ± 0.011	0.881 ± 0.039
Xception Head	0.460 ± 0.038	0.578 ± 0.017	0.559 ± 0.017	0.689 ± 0.010	0.629 ± 0.039	0.849 ± 0.082
Xception Abdomen	0.514 ± 0.015	0.589 ± 0.019	0.576 ± 0.023	0.698 ± 0.006	0.673 ± 0.011	0.904 ± 0.032
ResNet50 femur	0.492 ± 0.077	0.575 ± 0.007	0.514 ± 0.052	0.697 ± 0.002	0.693 ± 0.005	0.991 ± 0.015
ResNet50 head	0.548 ± 0.046	0.586 ± 0.014	0.680 ± 0.103	0.700 ± 0.005	0.698 ± 0.006	0.991 ± 0.008
ResNet50 abdomen	0.513 ± 0.062	0.571 ± 0.002	0.485 ± 0.003	0.696 ± 0.002	0.694 ± 0.003	0.996 ± 0.003

Results are presented as mean ± standard deviation. These values were obtained by threefold cross-validation. Columns include: AUROC: area under the receiver operating curve; PPV: positive predictive value; NPV: negative predictive value.

### Imaging data

Of the total 808 pregnant women included in the tabular data, each contributed with 3 US images of the third trimester (comprising the fetal head, abdomen and femur), totaling 2424 images. These were analyzed using a threefold cross validation, comprehending 1126 VD and 490 CS images for training and validation, and 563 VD and 245 CS images for testing the imaging-based models. Figures 1 and 2 and Table 2 present the imaging models’ performance in classifying VD vs CS. True delivery outcome served as the ground truth for training and testing. Overall, the best DL model for fetal US images was Inception, based on the same rationale as previously explained for the AdaBoost model. F1-score weighted and PPV for our test dataset were 0.594 ± 0.022, 0.580 ± 0.027 for femur (the best image model), and 0.590 ± 0.015; 0.571 ± 0.025 for abdomen, respectively. The head view’s F1-score weighted (0.587 ± 0.043) and PPV (0.565 ± 0.068) were the least helpful for mode of delivery prediction.

### Ensemble models

**Figure 3:**
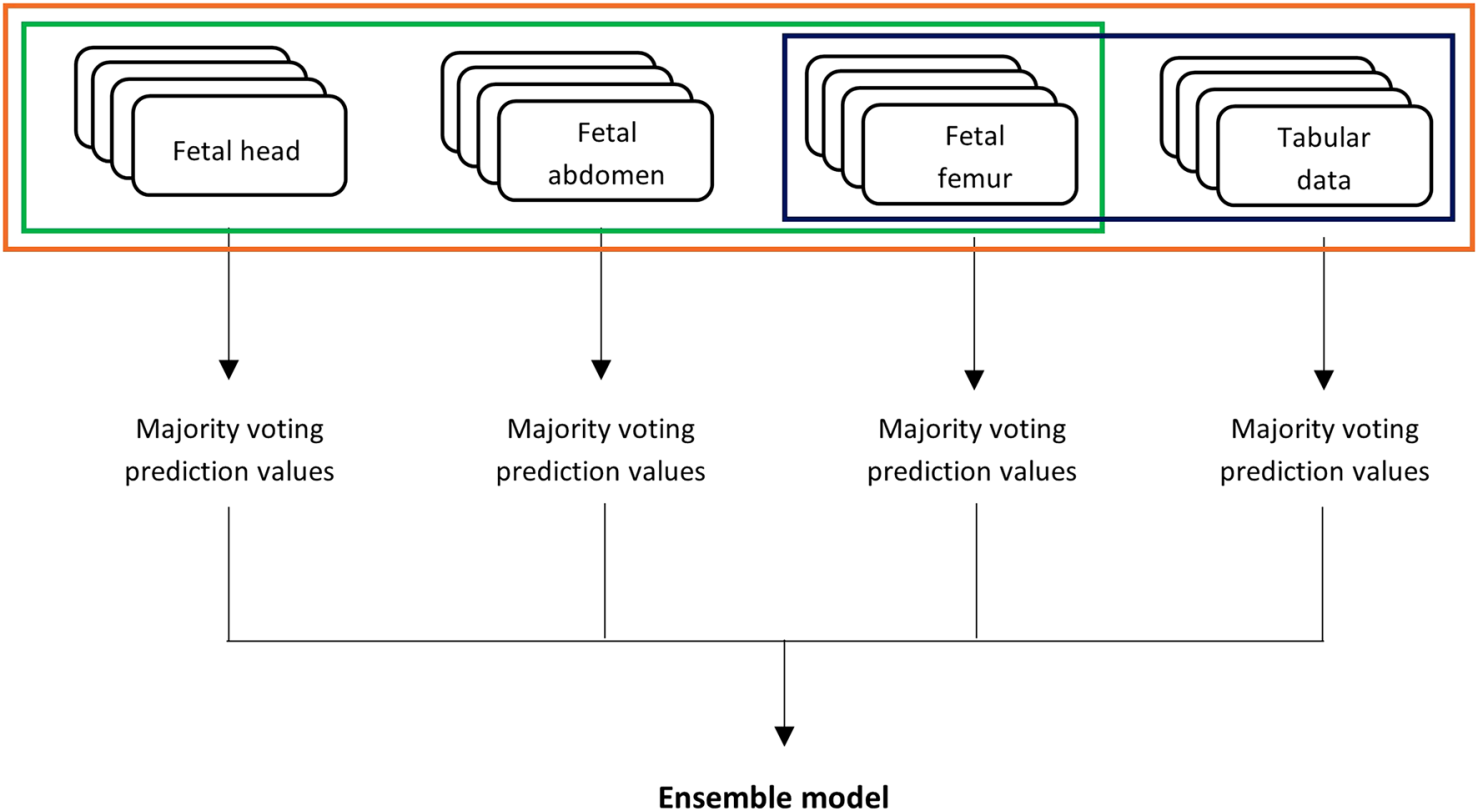
Process involved in the establishment of the ensemble models. The three image-based models (Inception head, abdomen and femur) were associated with the best tabular data model, AdaBoost in three different ways. Green box: Image-based model, using the CNN Inception model of the femur, abdomen and head; Orange box: AdaBoost tabular data model with Inception models of the femur, abdomen and head; and Blue box: the Final classification model, which consists of the AdaBoost tabular data model and the Inception model of the femur, which is the ensemble model which provides the best metrics.

**Figure 4:**
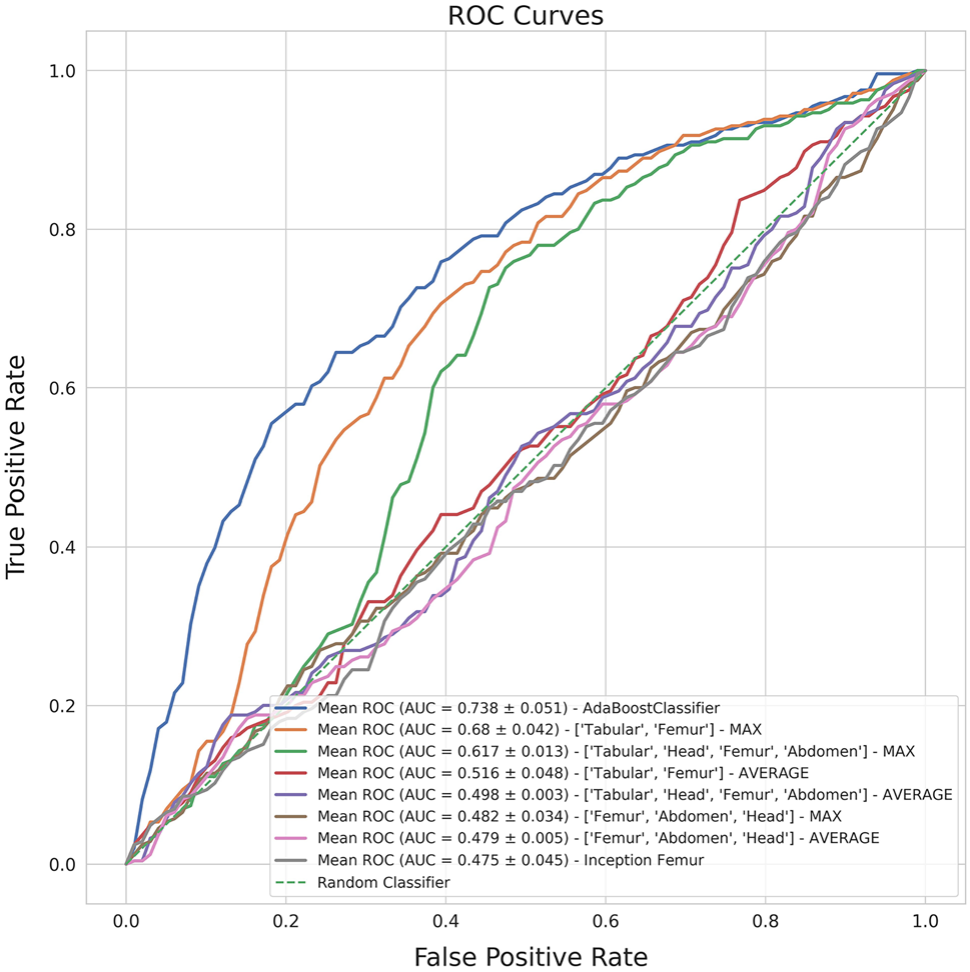
The ROC curves for prediction of mode of delivery for the ensemble models and their comparison with the ROC curves of the Adaboost and Inception femur models. ROC, receiver operating characteristic.

**Figure 5:**
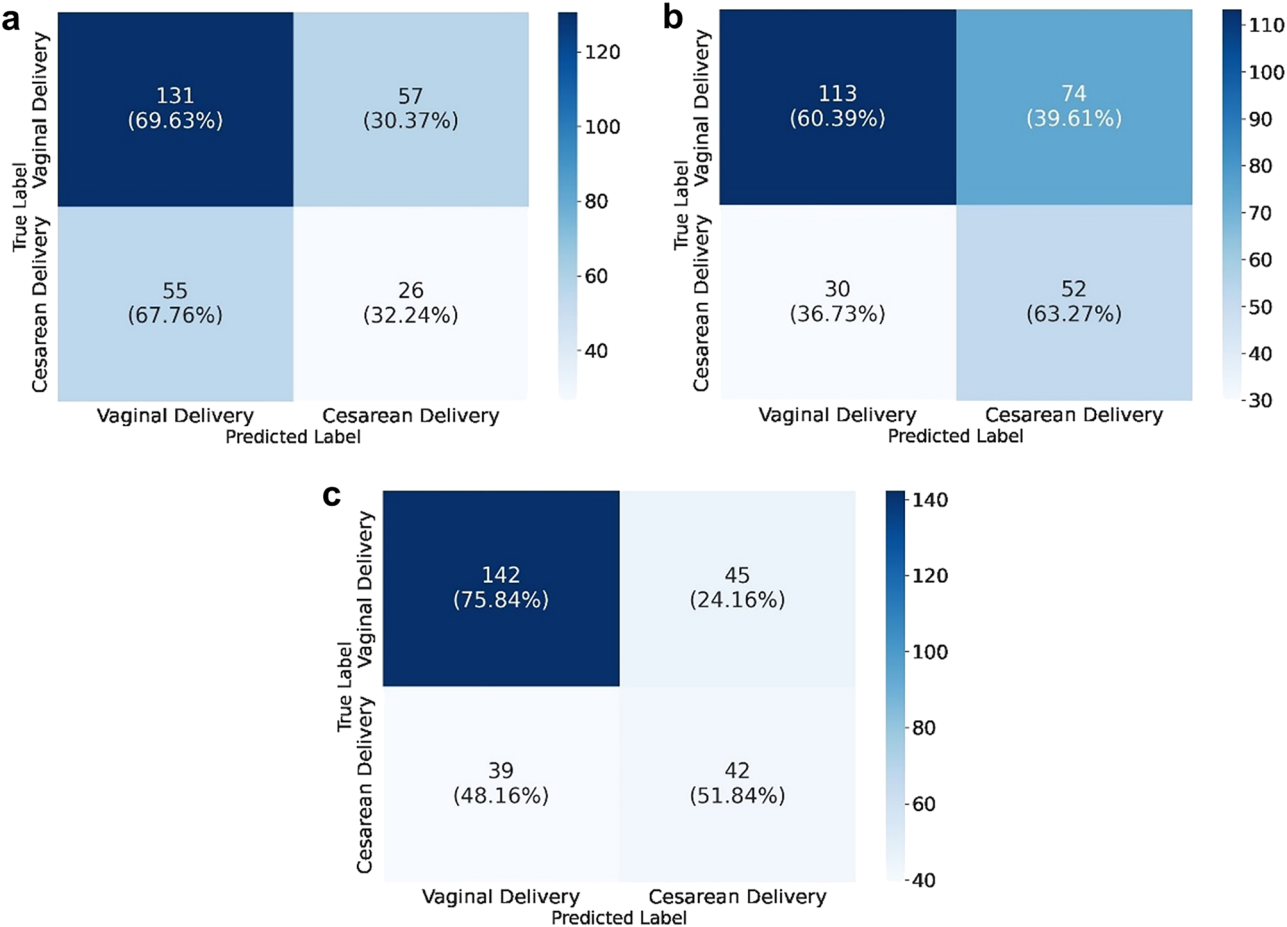
Confusion matrices on the following ensemble models: (**a**) image-based model, (**b**) AdaBoost and Inception models of the femur, head and abdomen (majority voting) (**c**) the final classification model (majority voting). Confusion matrix depicting in reading order from left to right, top to bottom: true-negative, false-negative, false-positive and true-positive rates.

Additionally, to test whether DL can improve mode of delivery prediction using multimodal imaging associated with tabular features, we implemented an ensemble of neural networks to provide classification on mode of delivery and compared their performance measures (see Fig. 3 for further explanation). We explored this approach by applying both average voting and majority voting strategies, the latter providing the best results, as shown in Table 3. The first ensemble model gathered the best US models of fetal head, abdomen and femur (image-based ensemble model), returning weak results in distinguishing VD vs CS, with a F1-score weighted of 0.584 ± 0.032 and a PPV of 0.585 ± 0.031. Marginally better results were shown by an ensemble model considering the previous three models and the AdaBoost model, providing a F1-score weighted of 0.628 ± 0.018 and a PPV of 0.675 ± 0.021 (see Table 3 and Fig. 4). The final classification ensemble model was the best ensemble model, aggregating the best tabular model (AdaBoost) and the best US image model (Inception femur). It achieved a F1-score weighted of 0.689 ± 0.042 and a PPV of 0.693 ± 0.038 (Table 3 and Fig. 4). It accurately predicted 75.9% VD and 51.9% of CS, corresponding to a 24.1% FP rate and 48.1% FN rate, with an overall accuracy of 68.7% (184/268; see Fig. 5c). The confusion matrix and respective AUROC of the final classification ensemble model are displayed in Figs. 4 and 5.

**Table 3:** Performance metrics of the best tabular, image model and ensemble models using majority voting and average strategies. The results are the averages and standard deviations of the threefold validation.

Model	AUROC	F1-score	PPV	NPV	Sensitivity	Specificity
Tabular data model (AdaBoost)	0.738 ± 0.051	0.736 ± 0.024	0.734 ± 0.024	0.790 ± 0.019	0.746 ± 0.022	0.867 ± 0.019
Image model (Inception femur)	0.485 ± 0.019	0.605 ± 0.015	0.596 ± 0.017	0.703 ± 0.006	0.668 ± 0.003	0.908 ± 0.022
Image-based model (Inception femur, abdomen and head) Max voting	0.470 ± 0.016	0.576 ± 0.016	0.572 ± 0.010	0.693 ± 0.007	0.582 ± 0.023	0.718 ± 0.040
Image-based model (Inception femur, abdomen and head) Mean voting	0.477 ± 0.001	0.578 ± 0.006	0.587 ± 0.087	0.698 ± 0.001	0.694 ± 0.003	0.991 ± 0.003
AdaBoost + Inception femur, abdomen and head Max voting	0.621 ± 0.053	0.638 ± 0.043	0.679 ± 0.036	0.792 ± 0.033	0.624 ± 0.045	0.623 ± 0.047
AdaBoost + Inception femur, abdomen and head Mean voting	0.500 ± 0.001	0.578 ± 0.006	0.587 ± 0.087	0.698 ± 0.001	0.694 ± 0.003	0.991 ± 0.003
Final classification model (AdaBoost + Inception femur) Max voting	0.705 ± 0.049	0.716 ± 0.028	0.719 ± 0.027	0.802 ± 0.018	0.714 ± 0.029	0.783 ± 0.024
Final classification model (AdaBoost + Inception femur) Mean voting	0.522 ± 0.016	0.608 ± 0.012	0.606 ± 0.011	0.705 ± 0.005	0.677 ± 0.004	0.924 ± 0.022

Results are presented as mean ± standard deviation. These values were obtained by threefold cross-validation. Columns include: AUROC: area under the receiver operating curve; PPV: positive predictive value; NPV: negative predictive value.

The best tabular data model (AdaBoost) provided an average accuracy improvement of 6.0% over the final classification ensemble model for CS prediction. However, concerning CS prediction, the final classification ensemble model correctly predicted 51.9% (vs 46.9%) of CS, with a FP rate of 24.1% (vs 13.3%), compared to the AdaBoost model. Therefore, the tabular data model missed 4 correct CS predictions (TP) over the final classification ensemble model, while avoiding 20 unnecessary CS (FP) (see Figs. 2a and 5c).

## Discussion

This study is the first to verify the feasibility of DL algorithms for the binary classification of mode of delivery after IOL using maternal and fetal electronic medical data and third trimester fetal US images. We developed ML models using tabular data and DL models for imaging data using transfer-learning methods. Our best-performing models were AdaBoost on tabular data, with a PPV 0.734; and the DL model Inception evaluating femur US images, with a PPV 0.580. Then, using ensemble-learning methodology, we developed various composite models, the best being based on AdaBoost and Inception US femur images, yielding a PPV of 0.693, matching the metrics of our best tabular model.

Recent studies use electronic medical information on maternal and fetal characteristics to construct prediction models regarding mode of delivery after IOL^26–28^. However, very few use ML with the same goal^2,29^. Also, several research studies explored third trimester US biometry planes for image segmentation^30,31^, image or plane classification^8,15,16^ and fetal biometry estimation^32,33^. US image classification has been mainly used for automatic fetal malformation detection^18,34^. However, to our knowledge, no study has yet reported the relation between third trimester US fetal plane imaging and mode of delivery outcomes after IOL.

In our study, maternal characteristics related to CS outcomes were compatible with literature findings (see Table 1). In our dataset, women submitted to unplanned CS were older, shorter, with higher BMI and lower Bishop scores compared with the VD group^6,26,35^. Fetal US characteristics such as EFW and fetal biometry measures were also significantly larger for fetuses who underwent CS, which is also compatible with literature^36–38^. However, FL showed no difference between groups. This is an exquisite finding because our best US image model uses femur images (see Fig. 1b and Fig. 2b). The explanation may lie in prenatal predictors of increased fetal adipose deposition, namely on the fetal thigh, which were found to be strong predictors of unplanned CS, compared to traditional fetal biometry and EFW^39–42^.

In fact, when analyzing individually, each DL image model underperformed, revealing the model’s difficulty in ascertaining which image features could aid in mode of delivery prediction (see Fig. 2 and Table 2). This was expected, for two main reasons: the first relates to the fact that DL models for object-detection and segmentation tasks are more accurate in identifying fetal standard biometry planes than classification models, because they can localize anatomical landmarks before classifying the plane, similar to human reasoning^10^; the other reason lies on understanding AI’s effectiveness in complementing clinical processes, since there is no study evaluating the accuracy of human evaluation of fetal third trimester US planes and their association with CS, probably due to an empirically unlikely association. Consequently, there is no practical way of evaluating if our metrics are reduced or if they can eventually supersede human intervention.

Regarding metrics for evaluation, our prediction model aims to counsel pregnant women undergoing IOL. Therefore, the main objective is to correctly advise those at high risk of CS and try to reduce the psychological and monetary burden of IOL on these women, as well as to confidently initiate and continue an IOL in women with a high probability of VD. Hence, the aim is to correctly identify true CS (TP) and avoid performing a CS on women who would have a VD (FP). As such, the most useful metrics in our study would be PPV, or the ratio of TP predictions to the total number of predicted positive observations; accuracy, or the proportion of correct predictions made by the model out of the total number of predictions; and sensitivity, defined as the ratio of TP predictions to all observations in the class^13^. That is why F1-score works better in our study than AUC, and because the presence of imbalanced data can influence the latter^43^. Hence, the DL models that provided the overall best PPV and F1-scores were the Inception group (see Table 2), which were consequently chosen for the ensemble models construction.

The first attempt on the ensemble model aggregated all Inception models (US images of the fetal head, femur and abdomen). Its performance showed a worse F1-score than the best Inception image model (femur) (0.584 vs 0.594) with a slightly superior PPV value (0.585 vs 0.580), probably because it aggregated the lower scores of the head and abdomen Inception models. Therefore, the next attempted ensemble model grouped all three Inception models and the Adaboost model. The latter probably influenced this ensemble positively, with F1-score and PPV of 0.628 (vs 0.584) and 0.675 (vs 0.585), compared with the image-based model, respectively. Since AI models can only account for information ‘seen’ during training, this model improved its performance by integrating imaging and electronic health record data^18^. Consequently, the last ensemble model, named final classification model, gathered the best tabular ML model and the best image model. Its performance was similar to the AdaBoost model, retrieving a F1-score of 0.689 (vs 0.736) and a PPV of 0.693 (vs 0.734). However, on a closer look at the confusion matrix, results show that the final classification model correctly predicted 51.9% of CS, more than the 46.9% rate of the AdaBoost model. On the other hand, the FP rates were more favorable for the AdaBoost model, showing a 13.2% rate (vs 24.1% on the final classification model). This trade-off between TP and FP can be explained by the difference in specificity (0.867 vs 0.758) and sensitivity (0.746 vs 0.689) of the AdaBoost model over the final classification one. Hence, we could infer that using DL femur US image models could help increase TP diagnosis at the expense of a marginal increase of FP cases^15^. As such, the model could be a useful clinical screening tool to distinguish women who are clear candidates for VD from those who have an extremely high risk of CS, or those who would benefit from a personalized mode of delivery planning. However, as emphasized in recent literature, AI tools should be used as an adjunct to the decision-making process, and the choices of the obstetrician and the pregnant woman should prevail when counseling on mode of delivery^19^.

This study has several strengths. To our knowledge, we present a novel database, comprising 2024 images from 808 fetuses, annotated for mode of delivery classification tasks using ground-truth information. This contrasts with most databases using similar images, which focus on image segmentation and plane classification and do not provide information regarding mode of delivery^8^.

The dataset accurately represents a real clinical setting, by being unbalanced and by using images collected retrospectively by various operators using various US machines. We opted not to use oversampling methods, i.e., to artificially increase the representation of minority classes and balance the dataset^13^. This would enhance our models’ performance but refrained from a real clinical scenario. Also, since our study used routine examination images suffering from speckle noise, low contrast, and variations of machines and settings, our models worked on their heterogeneity and complexities^8,16^. We argue that learning from diverse images enhances models’ adaptability and applicability in real-world scenarios by identifying consistent patterns and features^8,13,44^.

Data augmentation and use of clinical data along imaging data enhanced robustness and flexibility of the final models^16,17^. Finally, our model was thought to be plug and play and user-friendly without many restrictions to deal with real world clinical scenarios, allowing centers to upload deidentified images directly from workstations or hospitals to a cloud platform, with or without requiring additional patient data^17^.

The study is not without limitations. It is retrospective and uses data from a single center. This, especially for class imbalance databases such as ours, may have affected model training and testing and subsequently influenced model metrics, with emphasis on ROC curves^10,18^. Future developments may address this limitation by ensuring more CS images are available for successful binary classifier training. Also due to retrospective data collection, our model could not account for clinical or imagiological intrapartum variables such as fetal occiput position and engagement. The authors recognize the significance of these assessments, as supported by current research^24^.

The inclusion of numerous predictors in our sample size leads to concerns about overfitting. There might also exist a lack of a robust predictive accuracy assessment when using other data^14^. Therefore, we emphasize the importance of external validation as our next step, to assess the constraints of generalization and the possibility of multisite deployment of our model^17^. Finally, the results suggest it could be worth exploring data fusion approaches that combine into one model both streams of information, clinical data and image data.

In summary, this study proposed an ensemble AI model using US images of the fetal femur and maternal–fetal tabular data, yielding a relatively good performance. This is the first attempt to use this type of imaging data for mode of delivery prediction after IOL. The proposed model may become part of a promising tool in assisting mode of delivery counseling in clinical practice.

## Materials and methods

### Datasets

The dataset was retrospectively collected at the Obstetrics Department of University Hospital of Coimbra, a center with two sites (Obstetrics Department A and B), which are specialized maternal–fetal departments that manage thousands of births annually. Sample size was based on feasibility.

Tabular data included 2672 consecutive singleton vertex term pregnant women referred for IOL between January 2018 and December 2021. Other inclusion criteria were pregnant women ≥ 18 years of age and baseline Bishop score of ≤ 6. Planned CS, antepartum fetal demise, major fetal anomalies, and preterm births were excluded from analysis. EMR were analyzed, and, to ensure reliability of data, cases with no information on cervical examination at the time of admission were also excluded (n = 3). The final tabular dataset included 2434 deliveries.

The image dataset was collected based on the previous case selection, taking into consideration pregnant women attending our department for routine third trimester US evaluation. Images acquired during standard clinical practice were collected. Gestational age was computed from crown-rump length measurements on first trimester US^45^. Images were taken as a part of the Portuguese screening program, which recommends that the third trimester US should be performed between 30 and 32 weeks and 6 days of gestation^46^. Therefore, we decided to include a range of gestational ages from early third trimester (27 weeks) to 32 weeks and 6 days. Only third trimester US were considered for our visual computational model because first and second trimester US have specific goals that do not provide relevant information regarding mode of delivery planning. Of the 2434 subjects selected for tabular data, we excluded those who did not perform the third trimester US in our institution. Of the ones who did, we excluded those with missing US images, including only examinations which provided at least three US images per fetus (fetal head, abdomen and femur). This resulted in a final dataset of 808 deliveries (cases) and a total of 2024 US images.

Approval was obtained from the ethics committee of our center (protocol number CE-047/2022). Given the retrospective nature of the analysis, written informed permission was not required. Methods and results are reported in accordance with the TRIPOD guidelines^47^.

### Data collection

Regarding tabular data collection, maternal age, gravidity, parity, BMI, height, GA, Bishop score, IOL indications, mode of delivery, CS indications, intrapartum complications, neonatal birth weight and neonatal outcomes were among the features examined^2^. Data were collected on admission and at the onset of the first stage of labor, after pelvic examination and assessment of both mother and fetus.

Ten different US machines provided by three different manufacturers (Voluson E8, Voluson P8, GE Healthcare, Zipf, Austria; Xario 200G, Aplio a550, Aplio i700, Aplio a, Aplio 400, Aplio 500 Xario 200, Toshiba, Canon Medical, Netherlands, Europe; H540 Samsung) were used for examinations. The percentage and absolute number of images from GE, Toshiba and Samsung ultrasound machines were 3.0% (n = 24), 96.9% (n = 783) and 0.1% (n = 1), respectively. Images were taken using a curved transducer with a frequency range from 3 to 7.5 MHz. Twelve examiners with significant experience (5–35 years) in obstetric US conducted the examinations according to ISUOG guidelines^11^. All images were stored in the original Digital Imaging and Communication in Medicine (DICOM) format in our Astraia database and were retrospectively collected. This process was made to comply with minimal quality requirements, i.e., omitting those with an improper anatomical plane (badly taken or cropped).

Regarding IOL, the choice of cervical ripening methods varied according to WHO recommendations and Bishop scores. These included oxytocin infusion, prostaglandin analogues and extra‐amniotic balloon catheters^1,48,49^. Premature rupture of membranes was defined as membrane rupture at term before labor onset. Prolonged pregnancy was determined at ≥ 41 weeks^48^. The definition of labor was the presence of regular uterine contractions with cervical changes^50^. Our institution performs IOL according to ACOG and NICE recommendations^51,52^.

IOL indications were categorized into prolonged pregnancy, pregnancy hypertensive disorders (i.e., gestational hypertension; pre-eclampsia), gestational diabetes, pregnancy cholestasis, premature rupture of membranes, intrauterine growth restriction, and other fetal or maternal pathology (e.g.: thrombophilia)^1^.

The primary outcome was mode of delivery, with VD and CS as algorithm outputs. Secondary outcomes included IOL indication and method, time to delivery and maternal-neonatal outcomes^53^. Successful IOL was determined as VD after induction. CS indications were stratified between non-reassuring fetal heart rate and failed induction/labor dystocia^2^. IOL duration was the time from IOL initiation to delivery^1^. Failed induction referred to not reaching an active phase of labor within 48 h, with ruptured membranes and oxytocin for at least 12 h. Labor dystocia was defined as cessation of dilation or descent during labor^1,54^.

### Data processing

All images were saved in PNG (Portable Network Graphic) format, without compression to prevent quality loss. Each fetal subject’s head, femur, and abdominal photos were labeled with the appropriate classification for VD or CS. Every image was cropped to square proportions, respecting 537 × 537 pixels, centered in the ultrasound window, and then downsampled to 80 × 80 pixels. Through this procedure, the uniformity, comparability, and compatibility of image dimensions were ensured for DL techniques, which specifically call for square-dimensional images as input^55^. During the resizing process, all patient data was eliminated by cropping the image header, hence avoiding ethical concerns. The original ultrasound image’s margins were also cropped to remove unnecessary information.

Most prospective studies use images without caliper overlays in their models^8^. However, we chose to maintain caliper overlays, since these were burned to the image and could not be removed without altering the original image. We are also supported by other authors performing retrospective analysis, which state that the presence of caliper measurements had no discernible effect on their model’s ability to predict the primary result^17^.

### Study design and training and test sets

The ML models tested for tabular data were logistic regression (LR), multi-layer perceptron (MLP), random forest (RF), support vector machines (SVM), extreme gradient boosted trees (XGBoost) and AdaBoost classifiers^56,57^. The framework parameters of these models are off-the-shelf default scikit-learn and are available at both https://scikit-learn.org/ and our Github (https://github.com/PugtgYosuky/ensemble-prediction-delivery-type). Although an exhaustive study is beyond the scope of this article, we plan to do a more in-depth analysis for the best hyperparams in our future work. Before modeling, missing data were handled by simple imputation. Numerical features were transformed with RobustScaler method, removing data median and scales according to the quartile range. We used the interquartile range. Categorical features were “one-hot encoded” resulting in binary features.

Our source image dataset began with all fetal third trimester ultrasounds fitting the inclusion and exclusion criteria above (n = 808 studies), which correspond to 563 (69.7%) VD and 245 (30.3%) CS. Hence, the dataset’s composition exhibits an imbalance, similar to most real-world clinical scenarios^8^. We applied a stratified threefold cross-validation, similar to the work of Moon-Grady et al., thus the full image dataset was divided into three different datasets, using a proportion of two-thirds for training and a third for testing. Validation data used 30% of the training dataset. There was no subject overlap between the test, validation and training sets, to guarantee that no images from training cases were included in the test dataset. The models’ parameters were learned using the training set; the prediction error for hyperparameter tuning and model selection was estimated using the validation set. The test set evaluated the generalization error for each of the final models, to avoid potential overfitting to the training set^17,20^.

Images from the training dataset were used to train (1) a VD versus CS classifier, (2) construct an ensemble model between each image view (fetal head, abdomen and femur), (3) compare it with the tabular dataset model and (4) with an ensemble model that associates tabular clinical information and the imaging ensemble model.

### Model architecture and training parameters of image classification models

Convolution neural network (CNN) is a deep neural network architecture that excels at extracting features from unstructured data, such as medical images, allowing the automatic feature extraction of crucial information for the learning task^44,55^. We used three traditional CNN architectures to train on our data: Inception^58^, Resnet 50^59^ and Xception^60^. This study used transfer learning, a method developed to transfer fully trained model parameters from one large database to another to fine-tune training^17^. All networks underwent pre-training on the ImageNet Large Scale Visual Recognition Challenge (http://www.image-net.org/) and were trained using our training data^61^. To perform the intended binary classification task (VD vs CS), the top fully connected layers were replaced by a single layer of one neuron.

All nets were trained using Adam optimizer^12,13^. Nets were given a maximum of 1500 epochs to train before early stopping if the loss on the validation set did not decrease after 200 epochs^13^. Learning rates were 0,001 and batch size was 32^13^. Additionally, a set of image data augmentation approaches were applied to the images in the training dataset. These are required for the model to anticipate changes to imaging capture conditions, such as fetal position, amniotic fluid and placental tissue diverse locations and different zoom and focus adjustments^17,55^. It consisted of image random horizontal flips, rotations of up to 20° and random brightness up to 20°^15^. Several randomly augmented images were produced for every image in the training set and added to it, while keeping the non-augmented version^20^.

The networks were applied to the test images after training and frozen, producing a predicted confidence score between 0 and 1 for the CS outcome^20^. For both the VD and CS classifications, the network output yields likelihood percentages, both totaling 100%. The criterion for classifying a case as VD or CS was thus 50%, and the classification with the higher percentage was chosen as the outcome^15^. The top-performing models were recorded as the final output models (Table 2).

### Composite diagnostic classification

Different CNN algorithms extract image information in different ways. Ensemble learning is a method that uses multiple algorithms to achieve a better predictive performance than any individual algorithm^62^. We computed an ensemble model using our highest performing individual models evaluating US images of the fetal head, abdomen and femur (image-based ensemble model; see Table 2 and Figs. 1 and 2)^55^. We tested two types of voting strategies based on the outcome probabilities of each model, which included the mean and max voting. We found that the max voting strategy was the best voting strategy for our ensemble models (Table 3)^55^. For every model, a score of 50% and above was considered CS, and below 50% VD.

Image-based ensemble model was further compared with the tabular data classification model. Lastly, a final classification ensemble model was created by aggregating the probabilities of the image-based ensemble model and the best tabular data classification model. This model’s metrics were also compared with the metrics of the previously described models.

### Framework and training and prediction times

All models were implemented in Python using Keras (https://keras.io/, GitHub,2015) with TensorFlow (https:// www.tensorflow.org/) backend. Numpy (https://numpy.org), Matplotlib (https://matplotlib.org), and Scikit-learn (https://scikit-learn.org/stable/) were used to train and evaluate the models involved. Training was performed in a server with AMD RYZEN 5, 32 RAM and 2 GPUs Nvidia RTX 3080TI 12G. Prediction times per image averaged 3 ms for classification on a standard laptop (2.6-GHz Intel core, 16 GB of RAM).

### Model evaluation

AUROC, F1 score, sensitivity, specificity, PPV and negative predictive values (NPV) were used for model performance assessment. PPV and F1-score were selected as the most appropriate measures for the study’s model to predict mode of delivery, but AUROC measures were displayed nevertheless because they are a familiar metric in the medical field for model performance evaluation^63^. The model was also evaluated using a confusion matrix portraying TP, FP, TN and FN^55^. In the matrix, each row denotes an instance in the real label, and each column denotes an instance in the predicted label.

### Statistical methods

Categorical variables were shown as frequencies and percentages, and continuous variables were shown as mean ± SD. The χ2 test was used to compare categorical variables, and continuous variables were compared using two-sided Student’s t-test. The normal distribution of all continuous variables was assessed a priori using skewness, kurtosis, mean, standard deviation, and histogram curve. A *p*-value < 0.05 was considered significant.

### Ethics declarations

The Ethics Committee of the University Hospital Centre of Coimbra reviewed and approved the study on 22 April 2022 (CE-047/2022). Requirement for written informed consent was waived due to the retrospective, de-identified nature of the patient data.

## Data Availability

The data are not made publicly available for reasons of privacy, use in the development of other manuscripts and ethical restrictions. Nevertheless, the data are available from the corresponding author on reasonable request.

## Code availability

Code will be available upon publication.
