# Author Correction: Ensemble learning for fetal ultrasound and maternal–fetal data to predict mode of delivery after labor induction

**DOI:** 10.1038/s41598-024-69015-0

**Published:** 2024-08-06

**Authors:** Iolanda Ferreira, Joana Simões, Beatriz Pereira, João Correia, Ana Luísa Areia

**Affiliations:** 1 Faculty of Medicine of University of Coimbra, Obstetrics Department, University and Hospitalar Centre of Coimbra, Coimbra, Portugal; 2 Maternidade Doutor Daniel de Matos, R. Miguel Torga, 3030-165 Coimbra, Portugal; 3 Department of Informatics Engineering, Centre for Informatics and Systems of the University of Coimbra, University of Coimbra, Coimbra, Portugal; 4 Department of Physics, University of Coimbra, Coimbra, Portugal

Correction to: *Scientific Reports* 10.1038/s41598-024-65394-6, published online 03 July 2024

In the original version of this Article the author names, Iolanda Ferreira, Joana Simões, João Correia & Ana Luísa Areia were incorrectly given as Iolanda João Mora Cruz Freitas Ferreira, Joana Maria Silva Simões, João Nuno Gonçalves Costa Cavaleiro Correia & Ana Luísa Fialho de Amaral Areia.

The original Article has been corrected.

